# Supplementary material for: Patchy and widespread distribution of bacterial translation arrest peptides associated with the protein localization machinery
Source: Nat Commun. 2024 Apr 2;15:2711. doi: 10.1038/s41467-024-46993-3 (PMC10987492; doi:10.1038/s41467-024-46993-3)
Supplement: Supplementary file 3 — Description of Additional Supplementary Files [file 41467_2024_46993_MOESM3_ESM.pdf]

Description of Additional Supplementary Files

File Name: Supplementary Data 1
Description: Genome identifiers used in uORF searching

File Name: Supplementary Data 2
Description: A list of candidate monitoring substrate genes for *secA*

File Name: Supplementary Data 3
Description: A list of candidate monitoring substrate genes for *secDF*

File Name: Supplementary Data 4
Description: A list of candidate monitoring substrate genes for *yidC*

File Name: Supplementary Data 5
Description: A list of *E. coli* and *B. subtilis* genes which codes RAPP-like motif

File Name: Supplementary Data 6
Description: Strains used in this study

File Name: Supplementary Data 7
Description: Plasmids used in this study

File Name: Supplementary Data 8
Description: Primers used in this study

File Name: Supplementary Data 9
Description: DNA templates for in vitro transcription/translation reaction

File Name: Supplementary Software
Description: Scripts for bioinformatic analysis
