# Supplementary material for: Patchy and widespread distribution of bacterial translation arrest peptides associated with the protein localization machinery
Source: Nat Commun. 2024 Apr 2;15:2711. doi: 10.1038/s41467-024-46993-3 (PMC10987492; doi:10.1038/s41467-024-46993-3)
Supplement: Supplementary file 14 — Reporting Summary [file 41467_2024_46993_MOESM14_ESM.pdf]

nature portfolio

Corresponding author(s): Shinobu Chiba and Keigo Fujiwara

Last updated by author(s): Jan 22, 2024

Reporting Summary

Nature Portfolio wishes to improve the reproducibility of the work that we publish. This form provides structure for consistency and transparency in reporting. For further information on Nature Portfolio policies, see our Editorial Policies and the Editorial Policy Checklist.

Statistics

For all statistical analyses, confirm that the following items are present in the figure legend, table legend, main text, or Methods section.

n/a	Confirmed	
☐	☒	The exact sample size (*n*) for each experimental group/condition, given as a discrete number and unit of measurement
☐	☒	A statement on whether measurements were taken from distinct samples or whether the same sample was measured repeatedly
☒	☐	The statistical test(s) used AND whether they are one- or two-sided<br>*Only common tests should be described solely by name; describe more complex techniques in the Methods section.*
☒	☐	A description of all covariates tested
☒	☐	A description of any assumptions or corrections, such as tests of normality and adjustment for multiple comparisons
☐	☒	A full description of the statistical parameters including central tendency (e.g. means) or other basic estimates (e.g. regression coefficient) AND variation (e.g. standard deviation) or associated estimates of uncertainty (e.g. confidence intervals)
☒	☐	For null hypothesis testing, the test statistic (e.g. *F*, *t*, *r*) with confidence intervals, effect sizes, degrees of freedom and *P* value noted<br>*Give P values as exact values whenever suitable.*
☒	☐	For Bayesian analysis, information on the choice of priors and Markov chain Monte Carlo settings
☒	☐	For hierarchical and complex designs, identification of the appropriate level for tests and full reporting of outcomes
☒	☐	Estimates of effect sizes (e.g. Cohen's *d*, Pearson's *r*), indicating how they were calculated

Our web collection on statistics for biologists contains articles on many of the points above.

Software and code

Policy information about availability of computer code

Data collection	NCBI-genome-download script (version 0.3.3) were used for download genome sequences and annotation files from NCBI.
Data analysis	R 4.2.2 or 4.3.0 were used for data processing with packages: tidyverse v1.3.2 or v2.0.0, Biostrings v2.68.1, rtracklayer v1.60.0, tidysq v1.1.3, ggseqlogo v0.1, idpr v1.10.0, readxl v1.4.2, patchwork v1.1.2, ape v5.7.1, phytools v1.5.1, ggdist v3.3.0, rBLAST v0.99.2, and R.utils v2.12.2. BLAST+ v2.13.0 were used for homology search. MMseqs2 v14-7e284 were used for clustering. MAFFT v7.490 were used for sequence alignment. Signal sequences or transmembrane regions were predicted using SignalP v6.0, deepTMHMM v1.0.18, and TMHMM v2.0.

For manuscripts utilizing custom algorithms or software that are central to the research but not yet described in published literature, software must be made available to editors and reviewers. We strongly encourage code deposition in a community repository (e.g. GitHub). See the Nature Portfolio guidelines for submitting code & software for further information.

Data

Policy information about availability of data

All manuscripts must include a data availability statement. This statement should provide the following information, where applicable:

- Accession codes, unique identifiers, or web links for publicly available datasets
- A description of any restrictions on data availability
- For clinical datasets or third party data, please ensure that the statement adheres to our policy

Source data are provided with this paper.

Research involving human participants, their data, or biological material

Policy information about studies with human participants or human data. See also policy information about sex, gender (identity/presentation), and sexual orientation and race, ethnicity and racism.

Reporting on sex and gender	n/a
Reporting on race, ethnicity, or other socially relevant groupings	n/a
Population characteristics	n/a
Recruitment	n/a
Ethics oversight	n/a

Note that full information on the approval of the study protocol must also be provided in the manuscript.

Field-specific reporting

Please select the one below that is the best fit for your research. If you are not sure, read the appropriate sections before making your selection.

☒ Life sciences ☐ Behavioural & social sciences ☐ Ecological, evolutionary & environmental sciences

For a reference copy of the document with all sections, see nature.com/documents/nr-reporting-summary-flat.pdf

Life sciences study design

All studies must disclose on these points even when the disclosure is negative.

Sample size	Sample size was not calculated. Sample size was determined based on the variability of results.
Data exclusions	No data were excluded.
Replication	Data for Western blotting and toeprinting were obtained by at least two biological replicates. Data for b-galactosidase activity were obtained by three biological replicates and means, each data, and standard deviations were presented.
Randomization	Randomization was not performed because it was not applicable to the experiment.
Blinding	Blinding was not performed because the identity of the analyzed samples was known and blinding was not possible or applicable.

Reporting for specific materials, systems and methods

We require information from authors about some types of materials, experimental systems and methods used in many studies. Here, indicate whether each material, system or method listed is relevant to your study. If you are not sure if a list item applies to your research, read the appropriate section before selecting a response.

Materials & experimental systems

n/a	Involved in the study	
☐	☒	Antibodies
☒	☐	Eukaryotic cell lines
☒	☐	Palaeontology and archaeology
☒	☐	Animals and other organisms
☒	☐	Clinical data
☒	☐	Dual use research of concern
☒	☐	Plants

Methods

n/a	Involved in the study	
☒	☐	ChIP-seq
☒	☐	Flow cytometry
☒	☐	MRI-based neuroimaging

Antibodies

Antibodies used	anti-GFP antibody (Wako, 012-22541, Mouse Monoclonal, clone mFX75), anti-FLAG antibody (Sigma-Aldrich, F3165, Mouse Monoclonal, clone M2), anti-LacZa antiserum (Eurofins, Rabbit Polyclonal), Goat Anti-Mouse IgG (H + L)-HRP Conjugate (BIO-RAD, 170-6516) and Goat anti Rabbit IgG (H + L) HRP Conjugate (BIO-RAD, 170-6515)

Validation	The specificity of the anti-GFP, anti-FLAG and anti-LacZa antibodies were validated by Western blotting in this study, showing that the size of the proteins detected by the antibodies were equivalent to the size of target proteins synthesized in vitro.
